# Melatonin influences the early growth stage in *Zoysia japonica* Steud. by regulating plant oxidation and genes of hormones

**DOI:** 10.1038/s41598-021-91931-8

**Published:** 2021-06-11

**Authors:** Di Dong, Mengdi Wang, Yinreuizhi Li, Zhuocheng Liu, Shuwen Li, Yuehui Chao, Liebao Han

**Affiliations:** College of Grassland Science, Beijing Forestry University, Beijing, 100083 China

**Keywords:** Auxin, Plant molecular biology

## Abstract

*Zoysia japonica* is a commonly used turfgrass species around the world. Seed germination is a crucial stage in the plant life cycle and is particularly important for turf establishment and management. Experiments have confirmed that melatonin can be a potential regulator signal in seeds. To determine the effect of exogenous melatonin administration and explore the its potential in regulating seed growth, we studied the concentrations of several hormones and performed a transcriptome analysis of zoysia seeds after the application of melatonin. The total antioxidant capacity determination results showed that melatonin treatment could significantly improve the antioxidant capacity of zoysia seeds. The transcriptome analysis indicated that several of the regulatory pathways were involved in antioxidant activity and hormone activity. The hormones concentrations determination results showed that melatonin treatment contributed to decreased levels of cytokinin, abscisic acid and gibberellin in seeds, but had no significant effect on the secretion of auxin in early stages. Melatonin is able to affect the expression of IAA (indoleacetic acid) response genes. In addition, melatonin influences the other hormones by its synergy with other hormones. Transcriptome research in zoysia is helpful for understanding the regulation of melatonin and mechanisms underlying melatonin-mediated developmental processes in zoysia seeds.

## Introduction

Melatonin (MT, *N*-acetyl-5-methoxytryptamine), commonly known as a vertebrate neurohormone released by the pineal gland, is a tryptophan-derived metabolite. Melatonin is a versatile substance with diverse effects in various animal physiological processes. It was initially identified as signal in the process of circadian rhythmicity in animal systems^1^. It has been found to be involved in many physiological events such as sexual behavior, reproductive activity, immunological enhancement, and antioxidation^2–4^. Hernàndez-Ruiz proposed that melatonin was involved in plant physiology^5^. The possible influences of melatonin on physiological and cellular actions in plants have been widely explored. Though successive studies, the potential properties of melatonin have been widely demonstrated to be related to diverse aspect of the plant life cycle, such as vegetative growth, reproductive development, senescence and stress resistance^6^.

Plant hormones regulate seed germination through a complex network of hormones and other coordinated molecules that may be transferred from the external environment to the internal environment of the plant. Previous studies have suggested that melatonin can regulate the growth of various types of seedlings^7^. Low concentration of melatonin significantly promoted seed germination and seedling growth of stevia, while high concentration of melatonin inhibited it^8^. Melatonin can protect seeds against chilling stress and heat stress and improve seed viability and germination after heat or cold stresses^9^. It can also increase the tolerance of *Brassica rubrum* seeds and seedlings to copper at high concentrations. In addition, melatonin probably influences the level of auxin or shows auxin-like activity to activate seed growth. Melatonin can act as a lupin (*Lupinus albus* L.) cotyledon growth promoter with a similar mechanism to that of IAA^10^. Although the receptor-mediated gene expression with regard to melatonin has already been determined in mammalian systems, it is still unclear whether the melatonin receptors in plants point to a chemical response or a receptor-dependent response^6^.

Zoysia (*Zoysia japonica*), a widespread warm-season monocotyledonous perennial species, is one of the most important turfgrasses around the world^11^. It is utilized in golf courses, ornamental lawns, sports turf, city afforestation projects, soil and water conservation applications, windbreaks, sand-fixation projects and many other applications. Early seedling establishment is a vital stage that has an important impact on the establishment of lawns. Asexual propagation is the proper method for zoysia establishment, but it costs more than establishment from seed. In zoysia seed propagation, germination has been a major limitation^12^.The quality of seeds is the basic factor that assures grass production and turf establishment. It is essential to explore the fundamental mechanisms of seed germination in zoysia.

To investigate the effects of the application of melatonin on the germination of seeds under stress, the germination percentage of nontreated seeds and seeds treated with various melatonin concentrations was evaluated. In this study, melatonin-regulated transcriptome analyses have been applied to investigate the transcript-level changes and show the melatonin-related genes during germination. The new systemic transcriptome analysis in this study may not only provide more details of regulation patterns associated with melatonin mediation, but also set the stage for improving zoysia propagation through gene manipulation.

## Results

### Effect of melatonin treatment on seed germination rate

**Figure 1:**
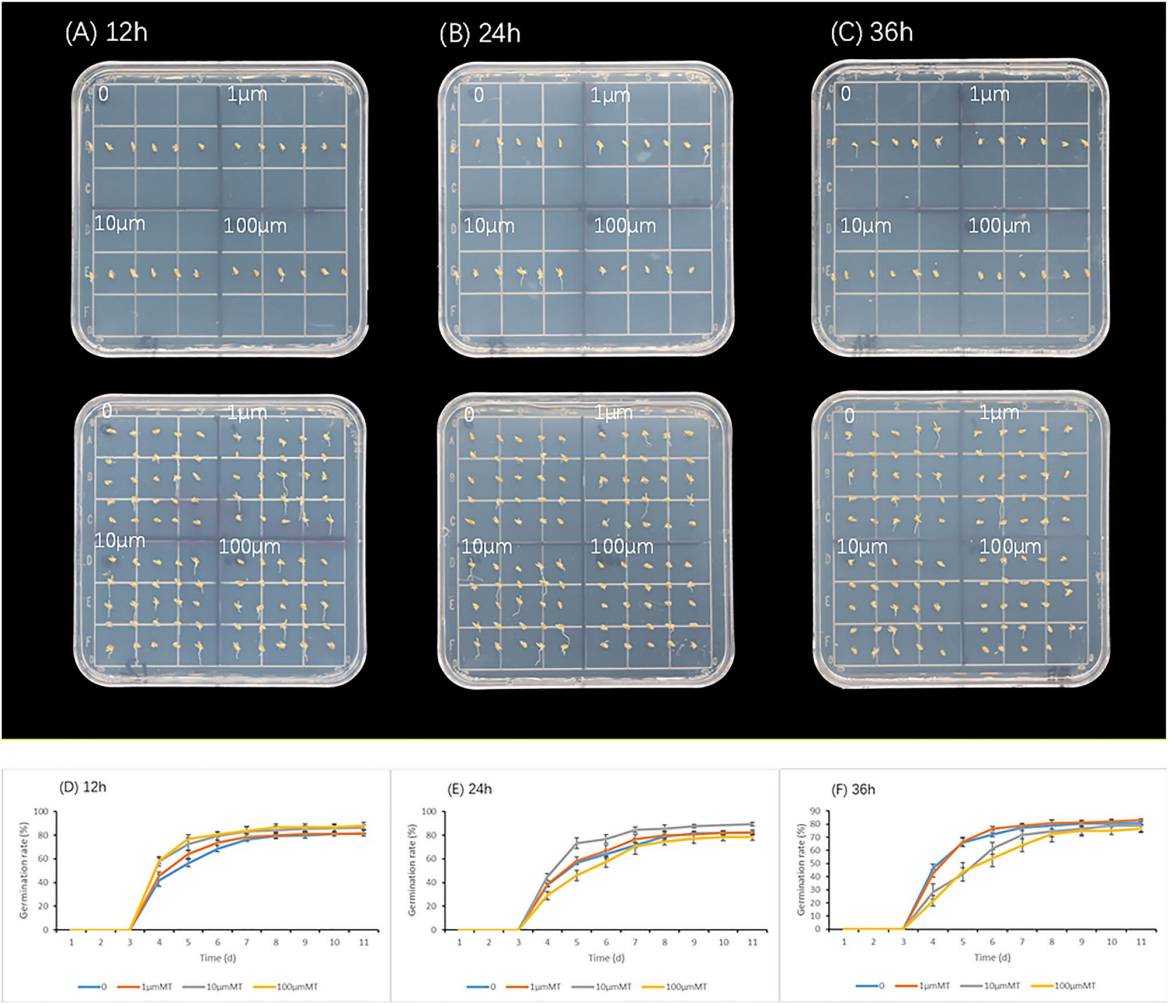
Seed of zoysia with different soaking times. Zoysia seeds were immersed in water and 1, 10, 100 μm melatonin solution for 12 (**A**), 24 (**B**), 36 (**C**) hours, respectively. The Germination rates of seeds zoysia soaked for 12 (**D**), 24 (**E**), 36 (**F**) hours were shown. Blue, red, gray, and yellow represent seeds treated with water, 1, 10, 100 μm melatonin, respectively. 24 h immersion in 10 μm melatonin solution was chosen as the treatment condition.

Germination assays of zoysia seeds with different concentrations of melatonin (1, 10, 100 μm) and different soaking times were conducted to select optimum concentration. At 96 h after imbibition, seeds began to germinate, the seeds of zoysia soaked in 10 μm melatonin for 24 h showed better performance (Fig. 1A–C). The daily germination rate within 11 days also showed that the germination rate of seeds soaked in 10 μm MT for 24 h was higher than seeds treated with other concentrations with the same soaking time (Fig. 1D–F). After immersion in water and 10 μm melatonin for 24 h, the germination rate of seeds was 82.666% ± 2.081% and 89.333% ± 1.528%, respectively (Fig. 1E). In this study, immersion of 10 μm MT for 24 h was selected as the treatment condition. The experimental results showed that melatonin treatment with specific concentration could increase the germination rate of seeds.

### RNA-Seq analysis and DEGs identification

**Figure 2:**
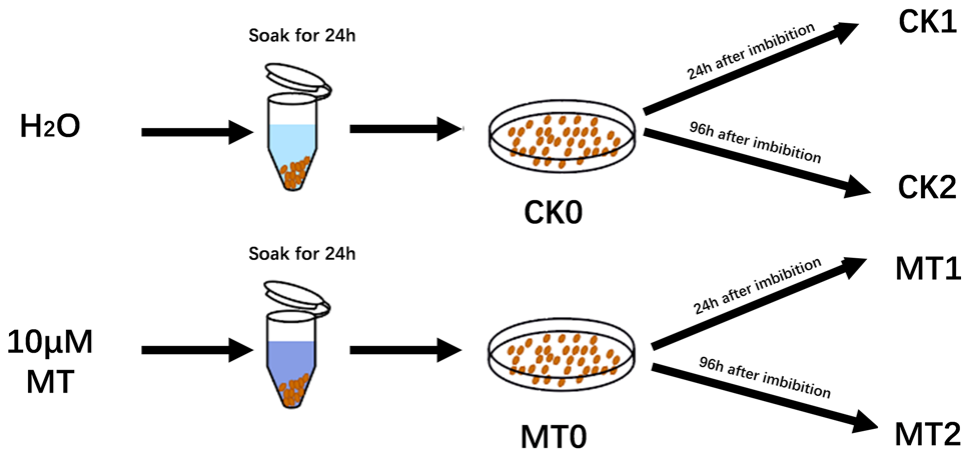
schematic of seed treatment. The seeds were immersed in water and 10 μm MT solutions for 24 h, and then placed on plastic petri dishes. At 24 and 96 h after imbibition, the water treated seeds were named CK1 and CK2, and the melatonin treated seeds were named MT1 and MT2.

The RNA was isolated from the melatonin-treated seeds and the control seeds (Fig. 2). RNA sequencing generated a total of 312.31 million raw reads from control sample and 305.22 million raw reads from melatonin treatment sample (Supplementary Table S1).

**Figure 3:**
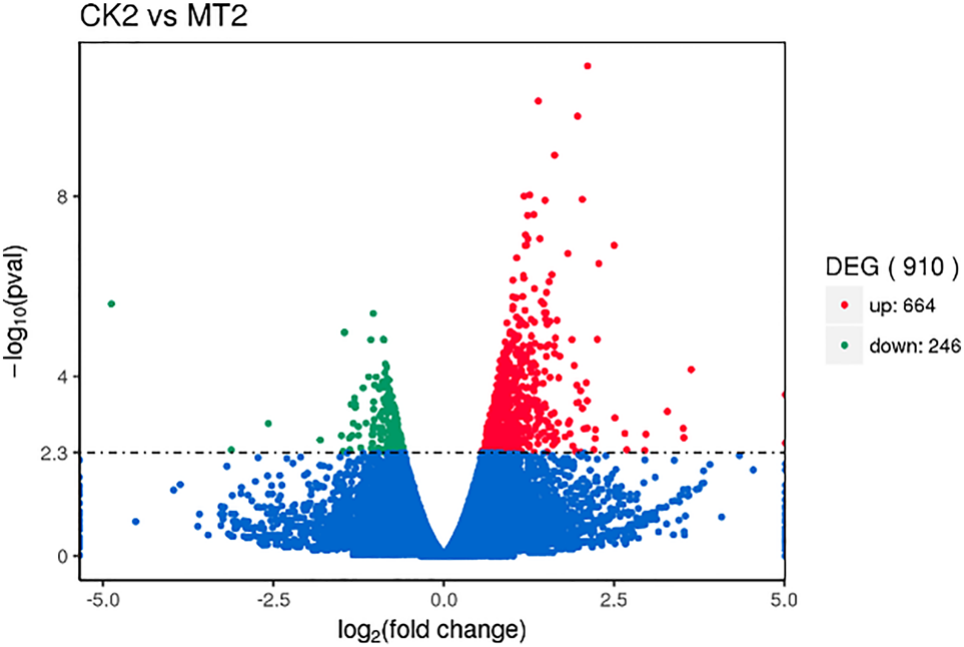
Volcano plots of DEGs in CK2 vs. MT2. Each dot represents one gene. Genes with no significant differences are represented by blue dots. DEGs were considered significantly different at a corrected q value < 0.005 and are represented by red dots (upregulation) and green dots (downregulation). Abscissas indicate gene expression fold changes in different samples; and ordinates represent genes with statistically significant differences in expression changes.

In CK2 vs. MT2, 910 DEGs were identified in the DEG analysis, with 664 upregulated genes and 246 downregulated genes (Fig. 3). There were large nonoverlapping gene groups between CK1 vs. CK2 and MT1 vs. MT2. The nonoverlapping genes that exists only in MT1 vs. MT2 were identified and abbreviated as T-MT in the following text, while nonoverlapping genes only in CK1 vs. CK2 were identified as T-CK (Supplementary Fig. S1). Several important DEGs (Zjn_sc00071.1.g00840.1.sm.mk, Zjn_sc00017.1.g06070.1.am.mk, Zjn_sc00012.1.g08710.1.am.mk, Zjn_sc00012.1.g08750.1.sm.mk, Zjn_sc00034.1.g02350.1.sm.mkhc, Zjn_sc00004.1.g14230.1.sm.mk, Zjn_sc00107.1.g00840.1.sm.mkhc) related to IAA response were identified in T-MT (Supplementary Table. S2).

**Figure 4:**
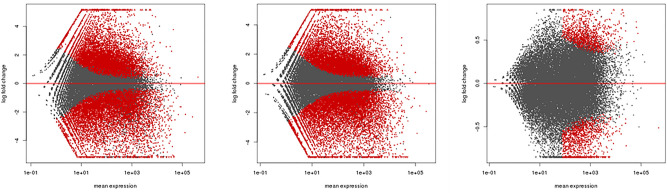
MA plots of differentially expressed genes (red points) and nondifferentially expressed genes (black points). The X axis is the mean expression, represented as gene expression abundances. The Y axis is the log 2 of the gene differential expression multiplier, which indicates the log ratio. (**a**) MA plot of DEGs in CK1 vs. CK2, (**b**) MA plot of DEGs in MT1 vs. MT2, (**c**) MA plot of DEGs in CK2 vs. MT2.

Comparing the differential genes between T-MT and T-CK could eliminate the effect of plant gene expression changes caused by growth and development, with the genes in the control samples that differed over time were removed. MA plots of the genes between combinations were generated to show the gene expression trends (Fig. 4). Different genes are activated by different conditions, a Venn diagram was drawn to show the overlapping relationship between the two combinations (Supplementary Fig. S3).

### Validation by qRT-PCR

A total of twelve genes were selected for quantitative RT-PCR assays (Supplementary Tables S3, S4). The expression patterns of the genes obtained by RNA-seq data were compared with those generated by qRT-PCR, and the results indicate that the expression trends of these 12 genes in RT-qPCR were consistent with those determined by RNA-Seq analysis.

### Gene ontology analysis and KEGG pathway analysis

**Figure 5:**
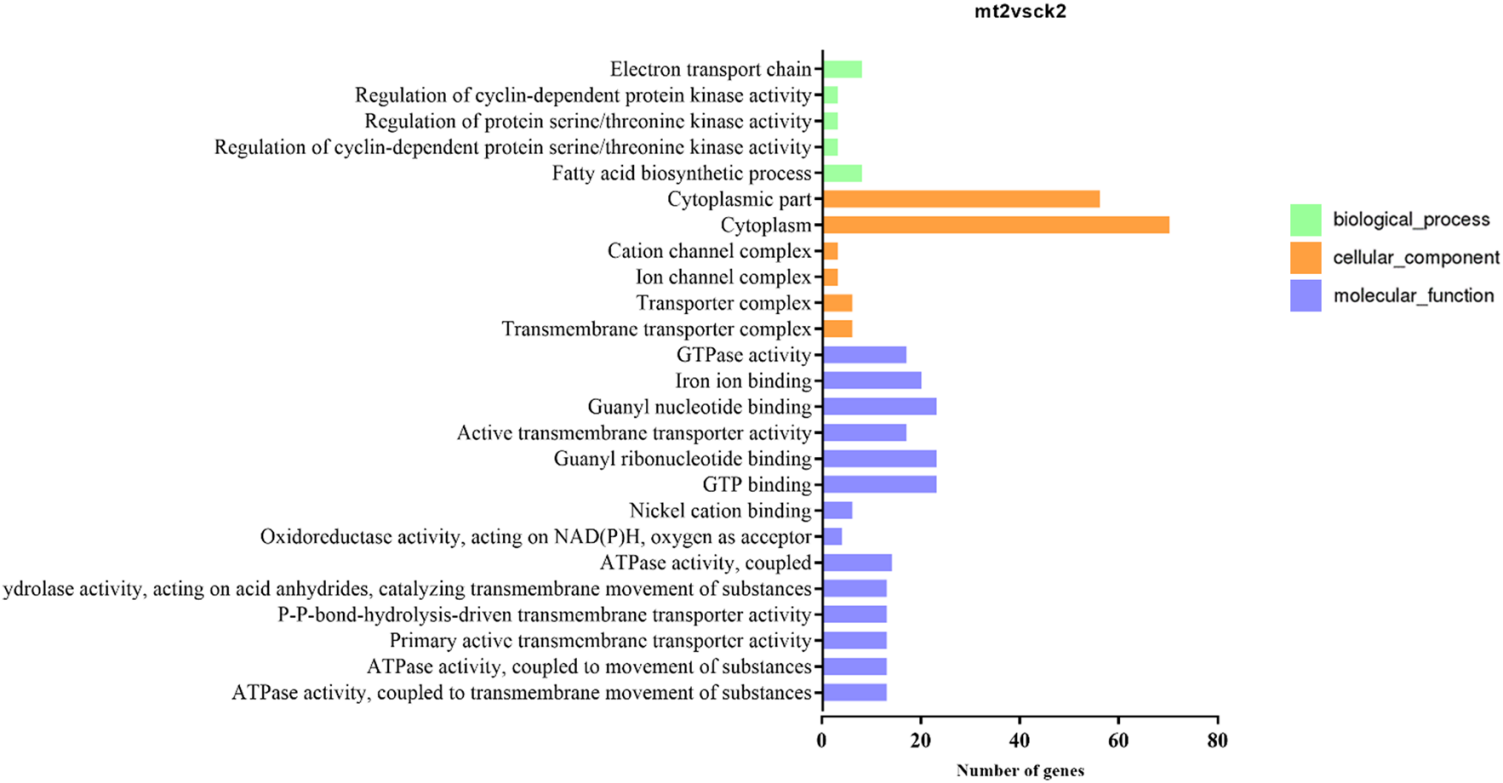
The most abundant gene ontologies including molecular functions, biological processes and cellular components in CK2 vs. MT2. “Cytoplasm (GO:0005737)” was the most abundant GO group, followed by “cytoplasmic part (GO:0044444)”, “GTP binding (GO:0005525)”, “guanyl ribonucleotide binding (GO:0032561)”, “guanyl nucleotide binding (GO:0019001)” and “iron ion binding (GO:0005506)”.

**Figure 6:**
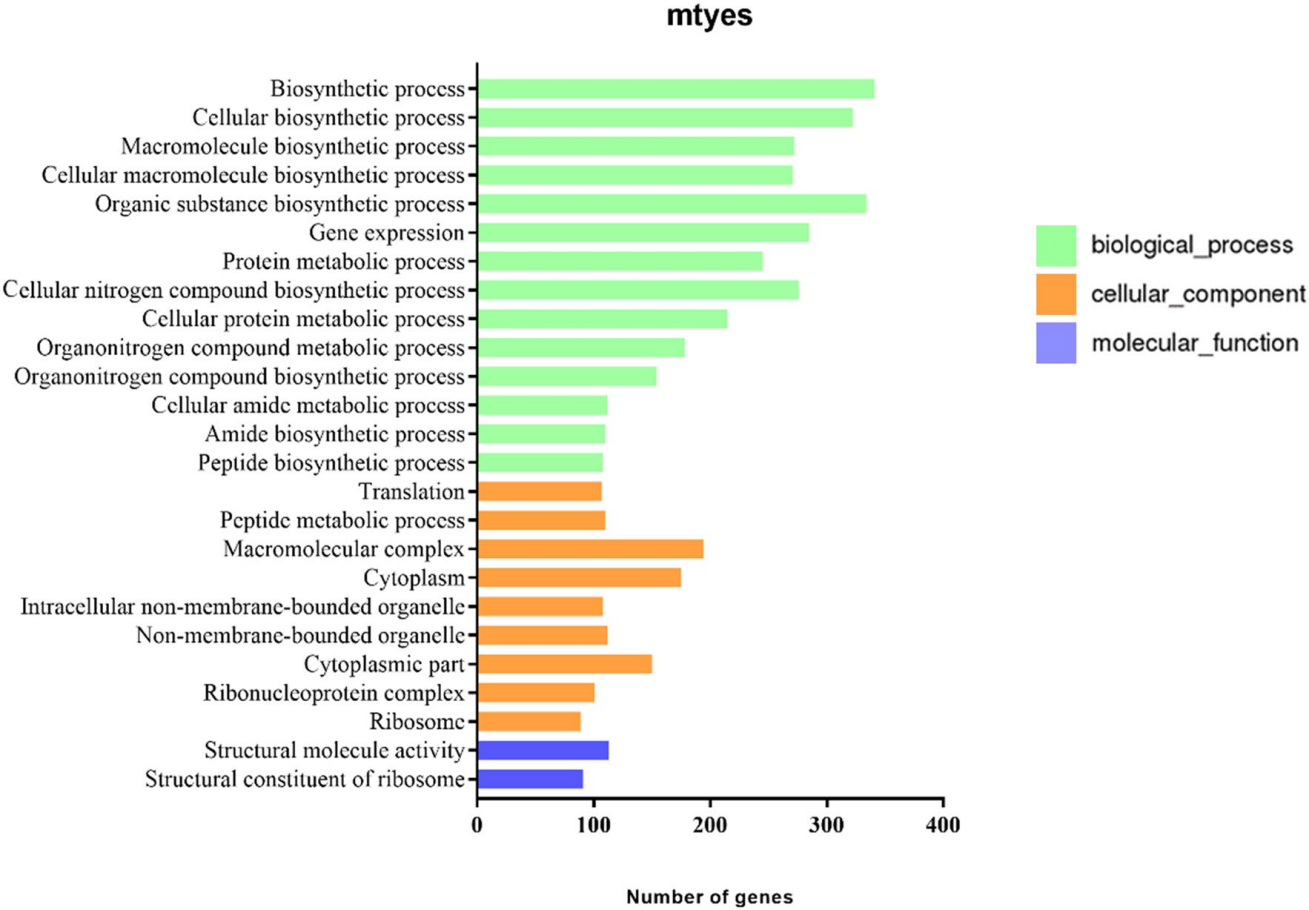
The most abundant gene ontologies in T-MT. In the biological process category, “biosynthetic process (GO:0009058)” and “organic substance biosynthetic process (GO:1901576)” were the most highly represented groups. For cellular component, the most-abundant groups were “macromolecular complex (GO:0032991)”. In terms of molecular function, “structural molecule activity (GO:0005198)” were overrepresented.

For data visualization and to acquire complete functional information, GO was performed to unify the gene attributes and classify the DEGs into presumptive functional groups. The DEGs were grouped into three categories, “biological processes”, “molecular function” and “cellular component”. In the three categories, 910 DEGs in CK2 vs. MT2 were grouped into 25 GO functional subcategory annotations and 2144 DEGs in T-MT were classified into 25 GO terms (Figs. 5, 6). These results showed that the biochemical and physiological processes of zoysia seeds treated with melatonin are quite different from those of the control group. These annotations provide valuable resources for the study of the specific processes, functions and pathways of melatonin in the growth and development of zoysia seeds.

**Figure 7:**
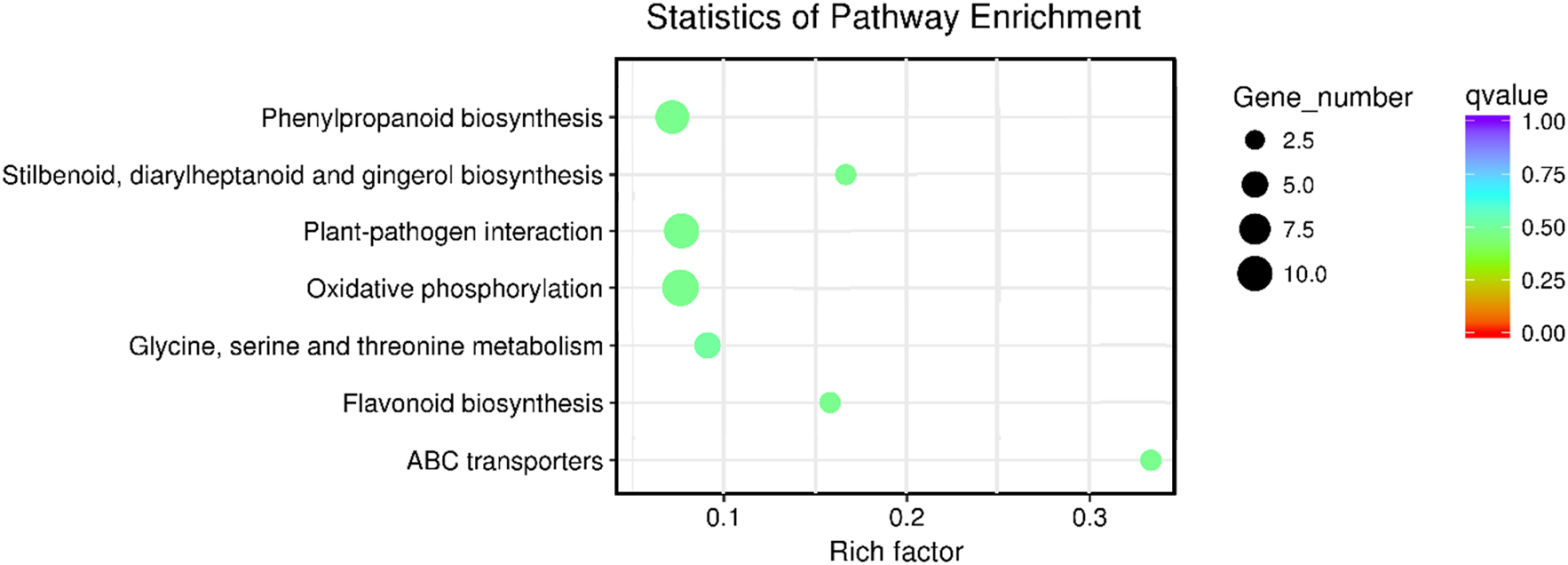
Kyoto Encyclopedia of Genes and Genomes pathway analysis of the DEGs in CK2 vs. MT2. The DEGs in CK2 vs. MT2 were enriched in 7 pathways. Genes corresponding to the categories “Oxidative phosphorylation”, “Plant–pathogen interaction”, “Phenylpropanoid biosynthesis”, “Glycine, serine and threonine metabolism”, “ABC transporters”, “Stilbenoid, diarylheptanoid and gingerol biosynthesis” and “Flavonoid biosynthesis” were enriched.

Pathway annotation allows the systematic analysis of intracellular metabolic pathways and gene functions, which can provide information on gene interactions. The DEGs in CK2 vs. MT2 were assigned into 20 pathways and enriched in 7 pathways. Important pathways involved in antioxidation include the “Oxidative phosphorylation”, “Phenylpropanoid biosynthesis”, and “Flavonoid biosynthesis” pathways. The results showed melatonin also has a significant regulatory effect on other metabolic processes, including amino acid (glycine, serine and threonine) metabolism and stilbenoid, diarylheptanoid and gingerol biosynthesis (Fig. 7).

### Analysis of protein interaction network

**Figure 8:**
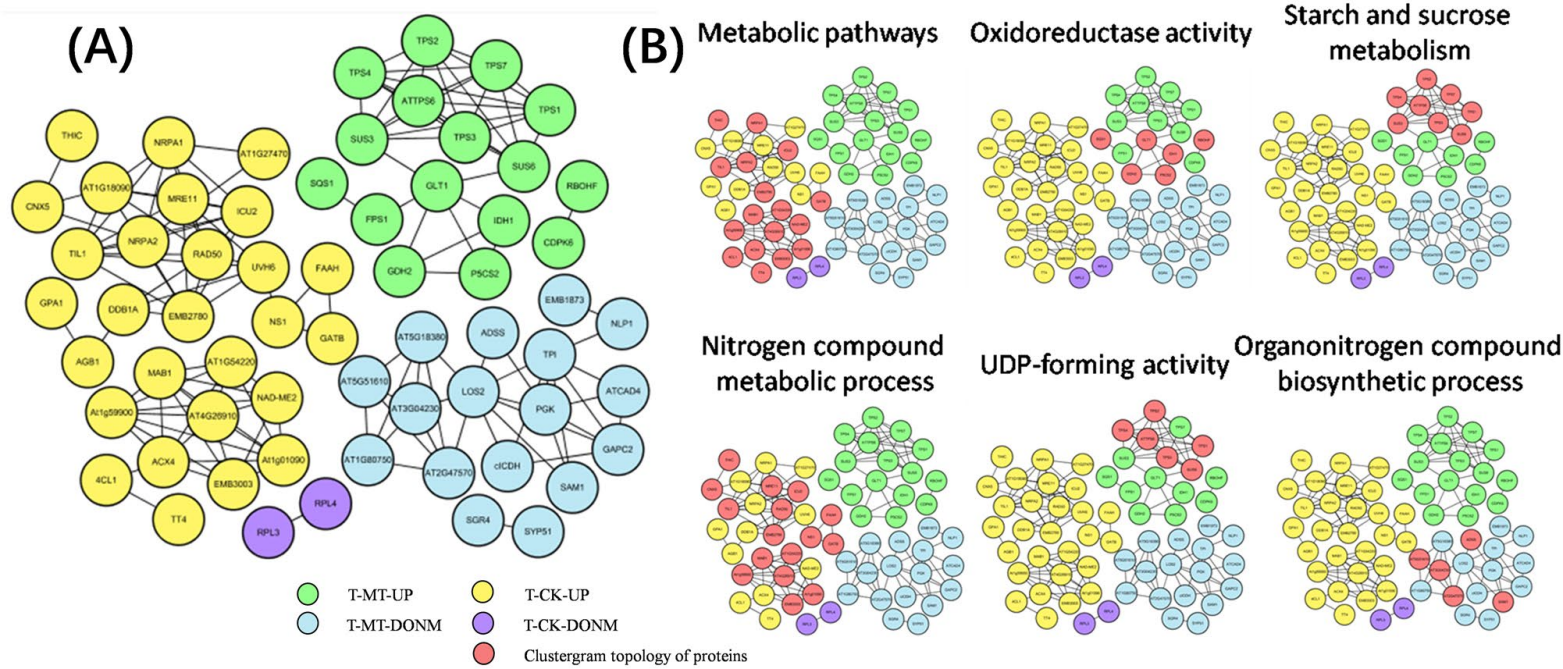
(**A**) Proteins from genes upregulated in T-MT (green), proteins from genes downregulated in T-MT (blue), proteins from genes upregulated in T-CK (yellow), and proteins from genes downregulated in T-CK (purple) identify a rich network of known and previously unknown proteins. Node titles correspond to the gene name, and connections represent interactions between proteins. (**B**) Clustergram topology of proteins (red) in selected functional categories or KEGG pathways.

A total of 63 differential genes were obtained by interaction network analysis. The network of protein interactions consisted of the proteins of upregulated and downregulated genes in the T-MT and T-CK groups, respectively (Fig. 8). Through GO and KEGG analysis, these proteins were found to be involved in multiple plant growth pathways. In the T-CK, 17 upregulated genes are involved in metabolic pathways, and 16 genes take part in nitrogen compound metabolic processes. The upregulated genes of T-MT are more related to oxidoreductase activity, starch and sucrose metabolism and UDP-forming (alpha, alpha-trehalose-phosphate synthase) activity. Several downregulated genes in the organonitrogen compound biosynthetic process are found in T-MT. This suggests that melatonin may affect the production of organic nitrogen in cells.

### Effects of exogenous melatonin on hormone content in seeds

**Figure 9:**
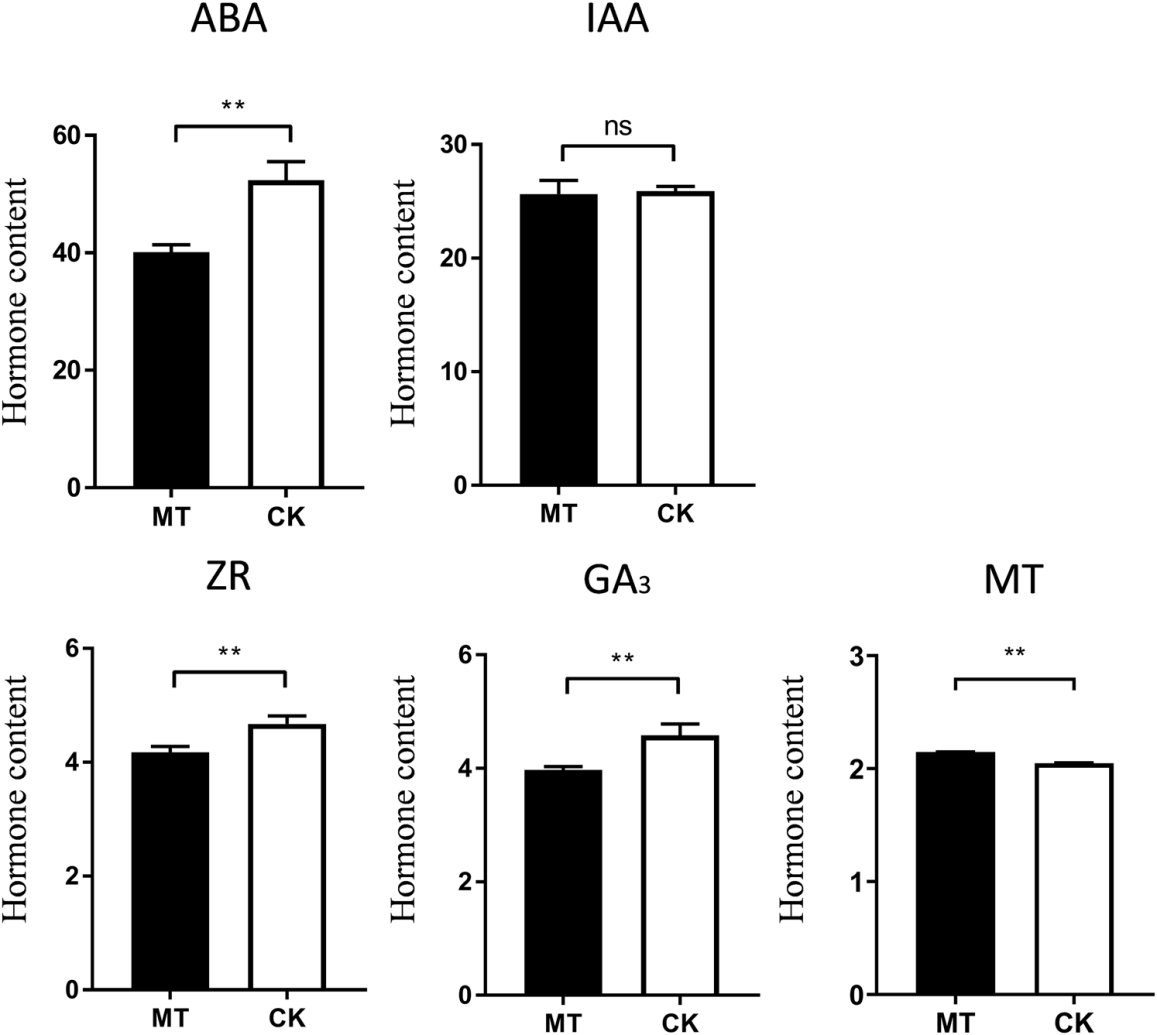
The contents of the plant hormones MT, IAA, ZR, GA3, and ABA were determined using icELISA. Values are means ± SD of three biological replicates. Bars indicate standard errors (n = 3). Student’s t test, **P < 0.01.

The application of exogenous melatonin significantly increased the content of endogenous melatonin (Fig. 9; Supplementary Fig. S4). After the application of exogenous melatonin, the content of endogenous melatonin in seeds was increased by 4.83% compared with that in seeds of the control group. In the tissues of seeds to which exogenous melatonin was applied, the IAA content was almost the same as that of the untreated seeds, and there was no significant difference (Fig. 9). The content of zeatin riboside in the tissues of seeds to which exogenous melatonin was applied was much lower than that in the control group, and the ZR (Zeaxanthin nucleoside) content in the seeds of the control group was higher than that in the experimental group by 12%. The concentration of GA3 (Gibberellin) in the seeds of the control group was higher than the GA3 concentration in the experimental group by 15.2%. After the administration of exogenous melatonin, the ABA (Abscisic acid) concentration in the seeds changed greatly, and the ABA concentration in the experimental group decreased by 23.4% compared with that in the control group.

### Effects of exogenous melatonin on antioxidant capacity of seeds

**Figure 10:**
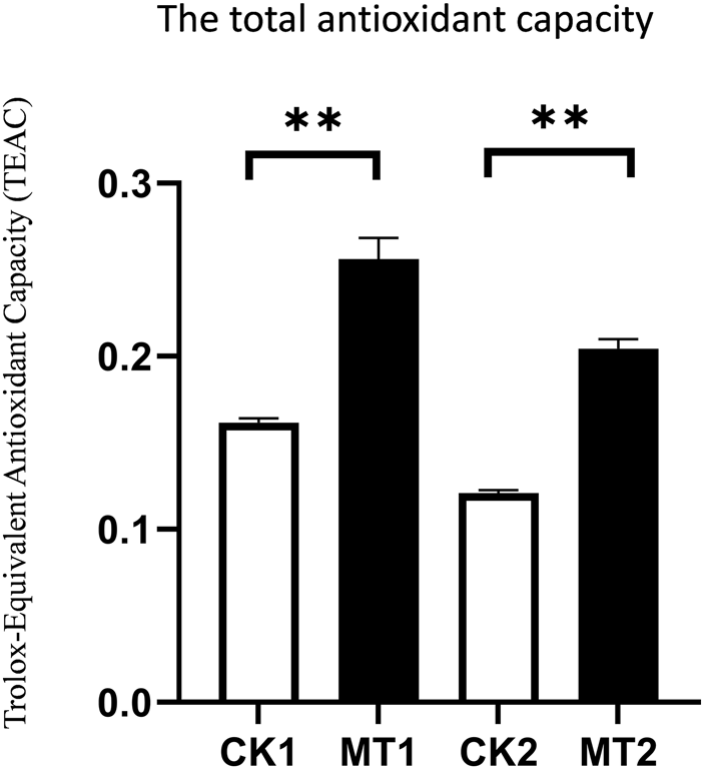
The total antioxidant capacity in water treated seeds was significantly lower than melatonin treated seeds. Values are means ± SD of three biological replicates. Bars indicate standard errors (n = 3). Student’s t test, **P < 0.01.

To investigate whether there was a difference in antioxidant capacity between Seeds treated with water and melatonin, The TAOC (Total antioxidant capacity) was investigated further using TEAC (Trolox-equivalent antioxidant capacity) as the reference (Fig. 10). TAOC results shows that the antioxidant capacity was lower in melatonin treated seeds than in the water treatment seeds. The experimental results showed that the antioxidant capacity of MT1 was 1.58 times that of CK1, while the antioxidant capacity of MT2 was 1.48 times that of CK2. The TEAC of water-treated seeds was significantly lower than melatonin-treated seeds, indicating that melatonin treatment can make seeds have higher antioxidant capacity and are less susceptible to oxidative damage.

## Discussion

Seed germination is an important stage in the life cycle of higher plants and a key limiting factor for turfgrass establishment through seed establishment. Studies have shown that melatonin is synthesized during cucumber seed germination, indicating that there is a correlation between its synthesis and seed germination^13^. With its remarkable antioxidative properties, melatonin behaves as a direct free radical scavenger and an indirect antioxidant to defeat organic radicals and reactive oxygen and reactive nitrogen species through cellular action. It has been surmised that melatonin existed in photosynthetic cyanobacteria as a strong antioxidant against toxic free radicals generated through photosynthesis in early evolution and played a subordinate role in the later stages of evolution^14^. The damage induced by free radicals is considered the reason for irreversible vigor loss in seeds^9,15,16^. Reactive oxygen species (ROS) must be strictly controlled to a low concentration by antioxidants to maintain the balance between antioxidant and prooxidative processes. Consequently, during the entire development of the seed, including germination and dormancy, seedling establishment, aging and death, antioxidants are critical^17^. Experiments have shown that melatonin can be used to maintain high vigor and germination in heat-stressed seeds, presumably due to its strong antioxidant capacity^18^.

According to the antioxidant capacity measurement results, melatonin-treated seeds have higher antioxidant capacity, which may be one of the important reasons why it affects the germination rate of zoysia seeds. It has been reported that melatonin alleviates plant oxidation by regulating metabolic changes^19^. The biosynthesis of melatonin is subject to an intricate regulation. Various anabolic and catabolic pathways are involved in this process. In the KEGG analysis of CK2 vs. MT2, the important pathways involved in antioxidation include “Phenylpropanoid biosynthesis” and “Flavonoid biosynthesis”. Flavonoids are considered primary antioxidants against irreversible oxidative damage. They may work through interactions with polar head groups of phospholipids at the lipid–water interface of membranes^20^. In addition, flavonoid is also related to many hormones; it may complement the function of endogenous auxin transport regulators to influence seed development, and increased ABA content may induce flavonoid biosynthesis via the MEP pathway^21^. Studies have shown that the genes involved in phenylpropanoid biosynthesis are significantly induced in the aging seeds treated with exogenous melatonin^22–24^. Phenylpropanoids can be activated by melatonin as effective antioxidants against abiotic stress^22,23^. In addition to oxidoreductase activity, the up-regulation of T-MT protein expression is also related to starch and sucrose metabolism. Metabolism of starch and sucrose is one of the most important physiological processes in seed development^25^. This suggests that melatonin-treated seeds may have a faster metabolism.

In addition to the effects from the antioxidant properties of melatonin, melatonin-treated seeds are also subject to a variety of physiological and metabolic effects at the seedling stage^14^. Hormones are important influencing factors for seed germination and growth. The hormone determination results showed that melatonin treatment inhibited cytokinin, abscisic acid and gibberellin in seeds but had no significant effect on the secretion of auxin in these stages.

IAA regulates cell elongation, division, and differentiation through the transcriptional regulation of specific genes to regulate the physiological and developmental processes of plants, including seed growth. Melatonin has shown possible action as a growth regulator in the same way as IAA in many studies^26^. The structure of melatonin is similar to those of tryptophan and IAA. They are both indole derivatives with a side chain at the C3 position^10^. Melatonin shares the beginning of its biosynthetic pathway with IAA; they are also found in similar concentration gradient in plant tissues. These common features may be the reasons that melatonin exhibits some auxin-like effects in plants and is regarded as a growth-regulatory signal or a regulator of reproductive development^27^. Some scientists believe that melatonin could hypothetically bind to auxin receptors and act directly as an auxin agonist^28^. However, there is no evidence or a detailed mechanism showing that melatonin manifests its auxin-like action by influencing IAA or acting directly as an auxin^6^. Considering the different characteristics of its side chain in comparison to that of IAA, melatonin does not fully meet the requirements of auxin activity in order to be a complete replacement for auxin. Park W J assumed that melatonin may perform IAA metabolic processes or affect the content of IAA^6^.

Comparative transcriptome analysis conducted on melatonin-treated *Arabidopsis thaliana* identified several DEGs^29^. In experiments with Arabidopsis, the GO functional and enrichment analysis results showed that DEGs were not enriched in GO terms associated with auxin response, such as “indole-3-acetic acid amido synthetase activity (GO:0010279)” and “auxin homeostasis (GO:0010252)”. The expression of two auxin response genes, *SAUR65* (*AUXIN UP RNA65*) and *RVE1* (*Resolvin E1*) were detected to be significantly reduced compared with controls. Researchers have speculated that MT was not affecting the expression of any auxin responsive genes except for these two genes 0^29^. Similar to the results in Arabidopsis, significant changes in several DEGs related to auxin response were observed under melatonin treatment, including four SAUR-like auxin responsive protein family genes and two ARF (ADP-ribosylation factor) family genes.

There are several and SAUR-like auxin-response protein family genes that changed in T-MT, which are the largest family of early auxin-response genes. Other hormonal and environmental factors also regulate SAUR (AUXIN UP RNA) gene expression. SAURs are thought to be a key gene family that regulates hormonal and environmental signals of plant growth and development^30^. Meanwhile, SAURS showed a rapid and transient response to IAA treatment^31^. After the treatment with melatonin, the expression levels of *ARF2* (*ADP-ribosylation factor 2*) and *ARF17* (*ADP-ribosylation factor 17*) in zoysia seed significantly decreased, while the expression levels in the control group did not change significantly. The process through which IAA participates in the growth and development of plants requires the transcription factor to control the expression of auxin response genes. Two types of transcription factors associated with auxin expression, ARF and the Aux/IAA proteins, are key regulators of auxin-mediated gene expression. The ARF family genes can promote or inhibit the auxin response genes through the combination of auxin action elements (AuxREs)^19^. As a regulator of auxin signaling, Aux/IAA proteins regulate auxin-mediated gene expression through their regulating effect on ARF transcription factor activity^32^. Researchers have speculated that *ARF2* inhibits cell division by regulating gene transcription downstream of cell growth- and senescence-associated signaling pathways^33^. ARF17 inhibits downstream expression of the GH3 family that encodes auxin-conjugating proteins, resulting in increased levels of IAA^34^. Although the application of melatonin had no significant effect on the content of IAA, it can influence the expression of regulatory factors and response genes during the course of IAA synthesis. Similar to other studies, melatonin appears to affect only a small number of hormone-related genes, but these genes play an important role in hormone action and activeity^29,35–37^. Our results suggest that melatonin may affect the activity and effects of IAA by regulating these genes (Supplementary Table S2), and may thus impact other hormones through its synergistic effect with other hormones.

Seed germination and development are complex physiological processes that are under the control of phytohormones. Both ABA catabolism and GA biosynthesis are considered crucial physiological mediation processes^38^. This study confirmed that the application of exogenous melatonin at 10 μm inhibited the hormone content of ABA and GA in zoysia. Studies have revealed that melatonin can act as a signaling molecule for ABA-derived catabolism and GA biosynthesis during seed development and regulation under high salinity, confirming the presence of melatonin-induced potential signaling pathways for ABA and gibberellin^13^. Some researchers believe that melatonin regulates seed germination by positively up-regulating GA biosynthesis and ABA catabolism^13^. Studies have also shown that melatonin can inhibit the content of GA3 at concentrations higher than a certain range^39^. The primary actions of ABA have been confirmed for a large number of species and include the promotion of protein and lipid synthesis for seed storage and the inhibition of germination when seeds are stored in water^40^. ABA also participates in and suppresses the embryonic to germinative growth transition phrase and the vegetative to reproductive growth transition phrase. The promotion of ABA degradation may be one of the possible mechanisms by which melatonin promotes the germination of zoysia seeds. Seed germination in many plants is accelerated by GA, which is considered to promote radicle protrusion and germination by impairing the mechanical inhibition of seed endosperm cells; GA acts as a requisite hormone against the germination constraints engendered by ABA-related embryo dormancy and seed coat limits^41,42^. The difference of GA hormone level in seeds after melatonin application may be related to melatonin concentration, application time and seed development stage.

## Methods

### Plant materials and sample preparation

Zoysia cultivar ‘Compadre’ seeds were purchased from the Hancock seed company (HANCOCK, USA). Plant use strictly followed institutional guidelines and governmental regulations. Mature seeds were surface-sterilized. Sterile seeds were soaked in a melatonin solution of 10 μm or in sterile water for 24 h in the dark at 24 °C ± 1 °C^43^. The water-treated seeds were the control. The seeds were soaked for 12, 24, and 36 h with three replications to identify the optimum duration of the melatonin treatment. After the final rinse, the seeds were each placed on plastic Petri dishes at 24 °C ± 1 °C. Seeds soaked in water and melatonin for 24 h were named CK0 and MT0, respectively. At 24 and 96 h after imbibition, the water treated seeds were designated as CK1 and CK2, and the melatonin treated seeds were designated as MT1 and MT2, respectively. All the treated seeds were quick-frozen in liquid nitrogen. The total RNA was isolated from the seeds of each treatment using Plant RNA Kit (OMEGA, USA).

### Library preparation for transcriptome sequencing

Electrophoretic profiles were generated with 1% agarose gels to monitor the samples for RNA degradation and DNA contamination. The purity of the RNA was checked using a NanoPhotometer spectrophotometer (IMPLEN, USA). A total amount of 3 µg RNA per sample was used as the input material for the RNA sample preparations. Sequencing libraries were generated using NEBNext Ultra RNA Library Prep Kit for Illumina (NEB, USA) following the manufacturer’s recommendations, and index codes were added to attribute sequences to each sample.

### Readings mapping and DEGs analyses

The library preparations were sequenced on an Illumina Hiseq platform. Reference genomes were downloaded from the genome website (http://zoysia.kazusa.or.jp/), and an index of the genomes was built using Bowtie v2.2.3. The gene model annotation files came specifically from this network. Differential expression analysis was performed using the DEGSeq R package (1.20.0). According to the Benjamini & Hochberg method, the corrected P value < 0. 005 and log2(fold change) > 1 were set as the DEG results filters to adjust the results for the P values^44^.

### Experimental validation of DEGs by qRT-PCR.

Quantitative real-time polymerase chain reactions (qRT-PCR) was used to validate the DEGs acquired by RNA-seq. Twelve genes involved in biosynthetic processes (GO:0009058) were selected for qRT-PCR, and the Zoysia beta-actin gene was used as a reference gene (GenBank accession No. GU290546). The results for the gene expression levels were further evaluated by their means with the corresponding standard deviations of three technical replicates.

### GO and KEGG enrichment analysis of DEGs

Gene Ontology (GO) analysis of DEGs were performed by the GOseq R package. In this process, gene length bias was corrected through the GOseq R package such that a P value of less than 0.05 was considered significantly enriched, and the statistical significance of the GO terms was determined. To identify DEGs with significantly enriched pathways, a DEG analysis using the Kyoto Encyclopedia of Genes and Genomes (KEGG) was performed^45,46^. A Q value of less than 0.05 indicates the statistical enrichment of DEGs in KEGG pathways. KOBAS software was applied to test the statistical enrichment^47^.

### Analysis of protein interaction network

The protein interaction database (http://string-db.org/) was used to analyze protein interaction network of DEGs^48^.

### Determination of MT, IAA, ZR, GA3, and ABA in seeds using icELISA

The extraction of the plant hormones MT, IAA, ZR, GA3, and ABA in CK2 and MT2, was carried out using a simplified indirect competitive enzyme-linked immunosorbent assay (icELISA)^49^. The concentrations of the standards and the OD at 490 nm of each sample were sequentially determined on Multiskan FC Microplate Photometer (THERMO SCIENTIFIC, USA).

The results were analyzed using logit curves. The logit value is calculated as follows:

$$\mathrm{Logit }\left(\frac{\mathrm{B}}{\mathrm{B}0}\right)=\mathrm{ ln} \frac{\frac{\mathrm{B}}{\mathrm{B}0}}{1 -\frac{\mathrm{B}}{\mathrm{B}0}}=\mathrm{ln}\frac{\mathrm{B}}{\mathrm{B}0-\mathrm{B}}$$

B0 is the color development value when the concentration is 0 ng/ml, and B is the color development value at other concentrations.

### Measurements of total antioxidant capability

Total antioxidant capacity (T-AOC) was assessed using rapid 3-ethylbenzthiazoline-6- sulfonic acid (ABTS) method (BEYOTIME, CHINA). 100 mg of each treated seed was accurately weighed and ground^50^. The extract was diluted 10 times for the measurement of antioxidant capacity according to the manufacturer’s instructions. Trolox solutions was diluted to 0.15, 0.30, 0.60, 0.90, 1.20, 1.50 mM, which was used to make standard curve. The Antioxidant Capacity of the sample was represented as Trolox-equivalent Antioxidant Capacity (TEAC)^51^.

## Supplementary Information

Supplementary Information 1.

## Data Availability

The datasets generated and analysed during the current study are available from the corresponding author on reasonable request.
