# Supplementary material for: Melatonin influences the early growth stage in Zoysia japonica Steud. by regulating plant oxidation and genes of hormones
Source: Sci Rep. 2021 Jun 11;11:12381. doi: 10.1038/s41598-021-91931-8 (PMC8196196; doi:10.1038/s41598-021-91931-8)
Supplement: Supplementary file 1 — Supplementary Information 1. [file 41598_2021_91931_MOESM1_ESM.docx]

**Melatonin influences the early growth stage in** ***Zoysia japonica* Steud. by regulating plant oxidation and genes of hormones**

**Di Dong^1^, Mengdi Wang^1^, Yinreuizhi Li^1^, Zhuocheng Liu^1^, Shuwen Li^1^, Yuehui Chao^1*^, Liebao Han^[[1]](#footnote-1)^***

Supplementary Table.S1 Data quality of RNA-Seq

| Sample name | Raw reads | Clean reads | clean bases | Error rate(%) | Q20(%) | Q30(%) | GC content(%) |
| --- | --- | --- | --- | --- | --- | --- | --- |
| CK1_1 | 46674152 | 42673158 | 6.4G | 0.02 | 95.55 | 89.53 | 61.31 |
| CK1_2 | 51104948 | 48367800 | 7.26G | 0.02 | 95.01 | 88.53 | 61.83 |
| CK1_3 | 57174922 | 53695618 | 8.05G | 0.02 | 95.75 | 89.83 | 61.01 |
| CK2_1 | 47443376 | 45029038 | 6.75G | 0.02 | 95.25 | 88.97 | 57.86 |
| CK2_2 | 51998302 | 50585598 | 7.59G | 0.02 | 95.96 | 90.28 | 56.71 |
| CK2_3 | 57923212 | 55652854 | 8.35G | 0.02 | 94.96 | 88.43 | 60.02 |
| MT1_1 | 58767038 | 56592634 | 8.49G | 0.02 | 96.04 | 90.4 | 60.49 |
| MT1_2 | 51391252 | 49366528 | 7.4G | 0.02 | 95.57 | 89.57 | 62.07 |
| MT1_3 | 55187866 | 53550182 | 8.03G | 0.02 | 95.74 | 89.87 | 58.95 |
| MT2_1 | 47601794 | 44393040 | 6.66G | 0.02 | 96.37 | 91.04 | 57.62 |
| MT2_2 | 48093768 | 46414578 | 6.96G | 0.02 | 96.01 | 90.34 | 58.43 |
| MT2_3 | 44179508 | 42740134 | 6.41G | 0.02 | 96.2 | 90.78 | 55.67 |

Supplementary Table.S2 List of DEGs associated with IAA response in T-MT

| Gene ID | Gene name | MT1_readcount | MT2_readcount | Log2ratio |
| --- | --- | --- | --- | --- |
| Zjn_sc00071.1.g00840.1.sm.mk | SAUR-like auxin-responsive protein family gene | 14.92467599 | 0.881175 | 4.0821 |
| Zjn_sc00034.1.g02350.1.sm.mkhc | ARF2, ARF1-BP, HSS, ORE14 \| auxin response factor 2 | 5156.218907 | 2220.143 | 1.2157 |
| Zjn_sc00004.1.g14230.1.sm.mk | ARF17 \| auxin response factor 17 | 98.25019824 | 45.18929 | 1.1205 |
| Zjn_sc00017.1.g06070.1.am.mk | SAUR-like auxin-responsive protein family gene | 0 | 8.734242 | - |
| Zjn_sc00012.1.g08710.1.am.mk | SAUR-like auxin-responsive protein family gene | 7.674860417 | 29.42759 | -1.939 |
| Zjn_sc00012.1.g08750.1.sm.mk | SAUR-like auxin-responsive protein family gene | 19.71307482 | 2.649467744 | 2.8954 |
| Zjn_sc00107.1.g00840.1.sm.mkhc | indoleacetic acid-induced protein 8 | 28.66979939 | 71.04098473 | -1.3091 |

Supplementary Table.S3 List of DEGs involved in “Biosynthetic Processes” as detected by RNA-Seq and qRT-PCR

| Gene ID | Gene name | Log_2_ratio | |
| --- | --- | --- | --- |
|  |  | RNA-seq | qRT-PCR |
| Zjn_sc00034.1.g06210.1.sm.mkhc | bZIP transcription factor family protein | -1.3805 | -1.5618 |
| Zjn_sc00107.1.g00840.1.sm.mkhc | indoleacetic acid-induced protein 8 | -1.3091 | -0.9454 |
| Zjn_sc00003.1.g01710.1.sm.mkhc | NAC domain containing protein 25 | -1.2547 | -0.8030 |
| Zjn_sc00013.1.g07460.1.sm.mk | AAA-ATPase 1 | 6.144 | 3.5675 |
| Zjn_sc00044.1.g05150.1.sm.mk | galactinol synthase 4 | 6.145 | 5.9424 |
| Zjn_sc00012.1.g06190.1.am.mk | glycerol-3-phosphate acyltransferase 5 | 5.7177 | 3.9423 |
| Zjn_sc00029.1.g02370.1.am.mkhc | NADH-dependent glutamate synthase 1 | 1.5227 | 3.2517 |
| Zjn_sc00036.1.g01750.1.am.mk | WRKY family transcription factor | 1.4598 | 6.8773 |
| Zjn_sc00040.1.g04580.1.am.mkhc | UDP-glucuronic acid decarboxylase 1 | 1.3804 | 1.5857 |
| Zjn_sc00071.1.g02790.1.am.mk | cellulose-synthase such as D2 | -5.7584 | -3.4643 |
| Zjn_sc00009.1.g11960.1.sm.mk | alpha-xylosidase 1 | -5.781 | -5.7260 |
| Zjn_sc00056.1.g02570.1.sm.mkhc | plasma membrane intrinsic protein 2 | -5.1833 | -2.4368 |
|  |  |  |  |

A total of twelve genes including TGA9, IAA8, NAC025, AATP1, GOLS4, GPAT5, GLT1, WRKY22, UXS1, CSLD2, XYL1, and PIP2B were selected for quantitative RT-PCR assays.

Supplementary Table.S4 Primers used for qRT-PCR validation

| Gene ID | Primer name | Primer [sequence](javascript:void(0);) (5’-3’) |
| --- | --- | --- |
| Zjn_sc00009.1.g00690.1.sm.mkhc | ZjACT-F | GGTCCTCTTCCAGCCATCCTTC |
|  | ZjACT-R | GTGCAAGGGCAGTGATCTCCTTG |
| Zjn_sc00034.1.g06210.1.sm.mkhc | TGA9-RT-F | GTGACCACCGATGATTACAGC |
|  | TGA9-RT-R | CACCCTCCAACGAACAGG |
| Zjn_sc00107.1.g00840.1.sm.mkhc | IAA8-RT-F | GTTACCGAAAGAACACCAT |
|  | IAA8-RT-R | CTGATTTCCCATTAGATTTG |
| Zjn_sc00003.1.g01710.1.sm.mkhc | NAC025-RT-F | CCGACAAGCCCATCCACA |
|  | NAC025-RT-R | CCCAGTCATCCAGCCTCAT |
| Zjn_sc00013.1.g07460.1.sm.mk | AATP1-RT-F | TCATCGACGGTCTGTGGTCC |
|  | AATP1-RT-R | GGCTCTTGGGCGTGAGGTT |
| Zjn_sc00044.1.g05150.1.sm.mk | GOLS4-RT-F | TGGAGGCACCCTGAGAACG |
|  | GOLS4-RT-R | ACGTCGCCAGACTCGGACA |
| Zjn_sc00012.1.g06190.1.am.mk | GPAT5-RT-F | CGGCAGATTACCCGAGTCCC |
|  | GPAT5-RT-R | CGAACGCCACGAGCATGAAG |
| Zjn_sc00029.1.g02370.1.am.mkhc | GLT1 -RT-F | GGGAACTGCCTTCTCGTA |
|  | GLT1 -RT-R | AACACCACTCGCAACAAC |
| Zjn_sc00036.1.g01750.1.am.mk | WRKY22-RT-F | CTGGCGACGGACAAGGGACA |
|  | WRKY22-RT-R | CGAAGCAGGCAGGAAGCAC |
| Zjn_sc00040.1.g04580.1.am.mkhc | UXS1-RT-F | AGCAACCGATGACTGTTTA |
|  | UXS1-RT-R | CTTTGGCTCCCAGTGTAG |
| Zjn_sc00071.1.g02790.1.am.mk | CSLD2-RT-F | CATTCCCAAGGCGTCCCA |
|  | CSLD2-RT-R | AACAGCCGCTCCGTCAGGT |
| Zjn_sc00009.1.g11960.1.sm.mk | XYL1-RT-F | CTTGAGTACAACGCTCACAGC |
|  | XYL1-RT-R | TGCCGAAGATGCCAAAGT |
| Zjn_sc00056.1.g02570.1.sm.mkhc | PIP2B-RT-F | CTACATCCTGGGCGACAACC |
|  | PIP2B-RT-R | GGCTCGGACTGCTGGAACA |


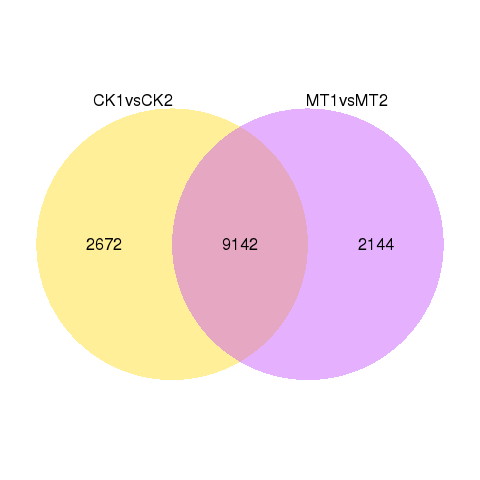


Supplementary Fig. S1 Venn diagram showed the genes in CK1 vs. CK2 and MT1 vs. MT2 that were expressed at different time points under different treatments. A total of 9142 differently expressed genes existed in the two combinations. There are 2672 genes in the T-CK, and 2144 genes in the T-MT.


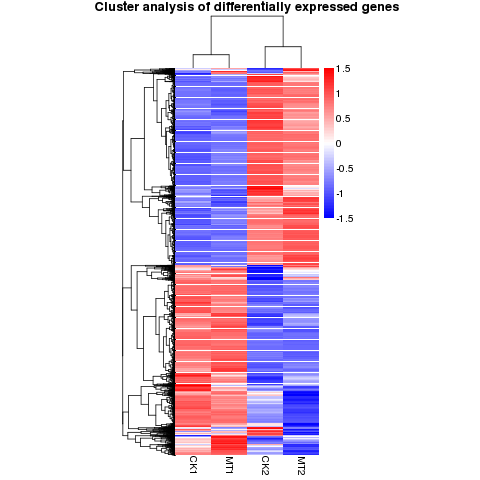


Supplementary Fig. S2 Hierarchical cluster analysis of gene expression in four transcripts based on log ratio RPKM data. Each column represents a treatment condition. Different colors denote different RPKM normalized log2-transformed counts (red and blue represent downregulation and upregulation, respectively).


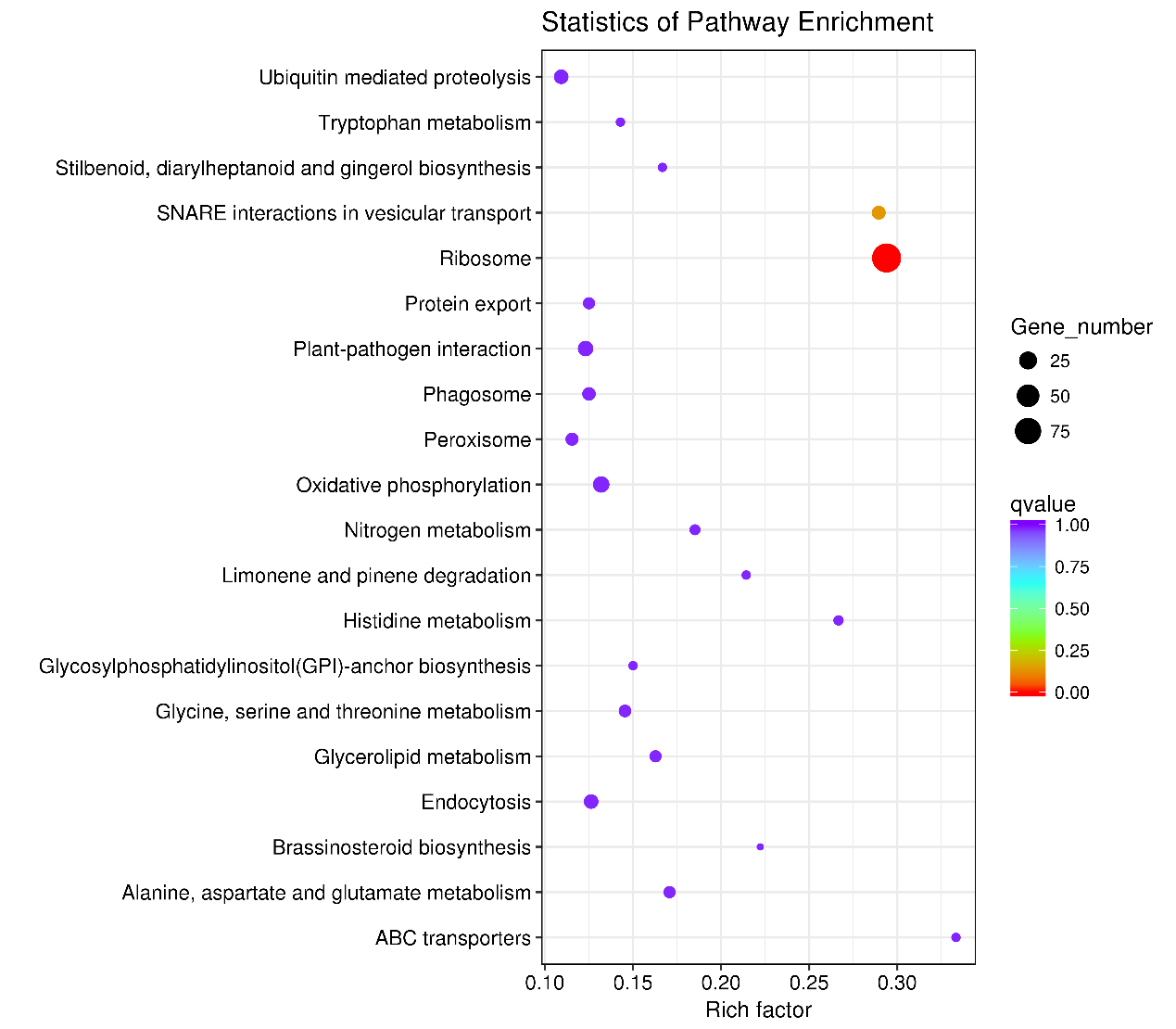


Supplementary Fig. S3 Kyoto Encyclopedia of Genes and Genomes pathway analysis of the DEGs in T-MT. DEGs in the T-MT were mapped to 50 pathways, and only 2 pathways were enriched. The DEGs were enriched in the “Ribosome” and “SNARE interactions in vesicular transport” pathways.

Supplementary Fig. S4 The contents of the melatonin were determined using icELISA. Data are means ± SD of three biological replicates. Bars indicate standard errors (n = 3). Different letters indicate significant differences between treatments at p < 0.05 level.

1. 1 College of Grassland Science, Beijing Forestry University, Beijing 100083, China [↑](#footnote-ref-1)
